# Combination strategies to overcome resistance to the BCL2 inhibitor venetoclax in hematologic malignancies

**DOI:** 10.1186/s12935-020-01614-z

**Published:** 2020-10-29

**Authors:** XiaoYan Yue, Qingxiao Chen, JingSong He

**Affiliations:** Bone Marrow Transplantation Center, Department of Hematology, The First Affiliated Hospital, School of Medicine, Zhejiang University, No. 79, Qingchun Road, Hangzhou, Zhejiang China

**Keywords:** BCL2, BCL-XL, Combination strategy, Hematologic malignancies, MCL1, Resistance, Venetoclax

## Abstract

Venetoclax has been approved by the United States Food and Drug Administration since 2016 as a monotherapy for treating patients with relapsed/refractory chronic lymphocytic leukemia having 17p deletion. It has led to a breakthrough in the treatment of hematologic malignancies in recent years. However, unfortunately, resistance to venetoclax is inevitable. Multiple studies confirmed that the upregulation of the anti-apoptotic proteins of the B-cell lymphoma 2 (BCL2) family mediated by various mechanisms, such as tumor microenvironment, and the activation of intracellular signaling pathways were the major factors leading to resistance to venetoclax. Therefore, only targeting BCL2 often fails to achieve the expected therapeutic effect. Based on the mechanism of resistance in specific hematologic malignancies, the combination of specific drugs with venetoclax was a clinically optional treatment strategy for overcoming resistance to venetoclax. This study aimed to summarize the possible resistance mechanisms of various hematologic tumors to venetoclax and the corresponding clinical strategies to overcome resistance to venetoclax in hematologic malignancies.

## Background

Apoptosis is a form of cell death, which is important in the development of the body, immune responses, and homeostasis. Apoptosis inhibition is also one of the characteristics of tumors, and the induction of apoptosis has become an important strategy for tumor therapy. The apoptotic pathway is regulated by the extrinsic and intrinsic pathways [1, 2]. The intrinsic apoptotic pathway is controlled by B-cell lymphoma-2 (BCL2) family proteins that can regulate the permeability of the outer mitochondrial membrane through protein interactions [3, 4]. The expression of BCL2 family proteins is usually dysregulated in hematologic malignancies [5]. A variety of tumor cells increased the expression of BCL2 family proteins through multiple mechanisms to ensure cell survival and proliferation, including chromosomal translocation, gene amplification, and downregulation/deletion of microRNAs that degrade BCL2 RNA [6, 7]. For example, BCL2 is usually overexpressed in multiple myeloma (MM) cells with t(11;14) [8]. The deletion of tumor suppressor genes microRNA-15 (miR-15) and microRNA-16 (miR-16) located in the 13q14 chromosome can also lead to increased expression of BCL2 [9, 10]. Therefore, targeting BCL2 can be used as one of the treatment strategies for tumors with high expression of BCL2.

Venetoclax, a BH3-mimetic, is a novel, oral, highly selective BCL2 inhibitor with high affinity for the BH3-binding groove of BCL2 [11]. It overcomes the apoptosis resistance and cell proliferation caused by the high expression of BCL2 in tumor cells. Previous clinical studies on venetoclax dose escalation showed 79% overall response rate (ORR) and 20% complete response rate (CR) in patients with chronic lymphocytic leukemia (CLL) having 17p deletion who were resistant to conventional chemotherapy. Within the dose range of 400–1200 mg, patients had similar treatment response rates and progression-free survival rate after 15 months [12]. In April 2016, venetoclax was approved by the United States Food and Drug Administration (FDA) as a monotherapy for treating patients with relapsed and refractory (RR) CLL having 17p deletion [12]. In addition, venetoclax has also shown significant activity in many hematologic malignancies, such as acute myeloid leukemia (AML), non-Hodgkin's lymphoma (NHL), MM, and so forth, not just in CLL [12–15]. Clinical studies showed venetoclax to be effective in patients with NHL and MM, with the efficacy of monotherapy ranging from 10 to 50% in patients resistant or intolerant to conventional chemotherapy or immunochemotherapy [8, 13]. In patients with AML, venetoclax monotherapy could benefit patients with refractory/recurrence or who were not suitable for standard chemotherapy-induced therapy; the efficiency of venetoclax could be further improved by combining with hypomethylating agents (HMAs) or low-dose cytarabine (LDAC) [16–18].

Nevertheless, venetoclax could still cause therapeutic resistance. The overexpression of the other anti-apoptotic proteins in the BCL2 family, such as myeloid cell lymphoma-1 (MCL1) and BCL2 like 1 (BCL2L1, also known as BCL-XL), mediated by various mechanisms has been experimentally demonstrated to lead to resistance to venetoclax [17, 19]. Therefore, some studies suggested that the ratio of BCL2 family protein expression rather than BCL2 expression could predict the efficacy of venetoclax [5]. According to the mechanism of venetoclax resistance in hematologic malignancies, drugs with different mechanisms are selected in hopes of overcoming the resistance to venetoclax, which can be used in combination with venetoclax. Common combination drugs include small-molecule kinase inhibitors (ibrutinib, sunitinib, idelalisib, and so forth), HAMs (azacytidine and decitabine), anti-CD20 monoclonal antibodies (rituximab and obinutuzumab), and highly selective MCL1 and BCL-XL inhibitors. This study aimed to summarize the possible mechanisms of resistance to venetoclax in a variety of hematologic malignancies and combination strategies for overcoming resistance to venetoclax in recent years.

## Resistance mechanisms of venetoclax

**Fig. 1:**
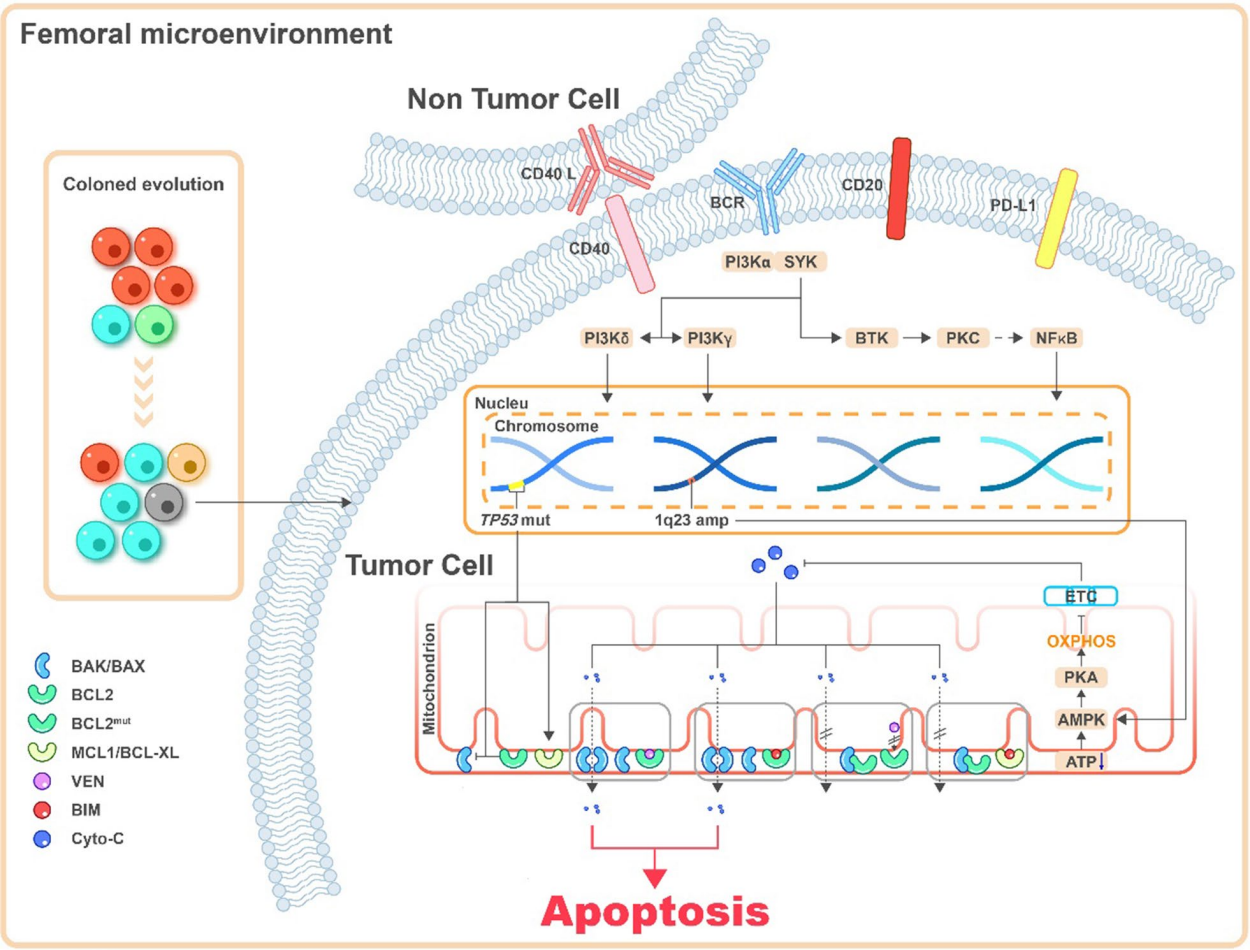
Potential mechanisms of venetoclax resistance. Ventoclax recognizes the BH3-biding groove of BCL2 and eases BAK/BAX complex formation, releasing cytochrome c from mitochondria and promote tumor cell apoptosis. Mutation of BCL2 changes protein conformation and impedes venetoclax binding to its target thus against its pro-apoptic effect. Also, genetic alterations such as mutation of *TP53* and amplification of 1q23 combined with running out of ATP on mitochondria membrane lead AMPK/PKA pathway aberrantly activating, diminishing the permeability of mitochondria membrane and inducing venetoclax resistance. Interact with non-tumor cells in surrounding microenvironment though membrane molecules activate multiple signaling pathways including NF-κB and PI3K/AKT, which upregulating the anti-apoptic proteins and relseasing varity of inflammatory cytokines. Gene mutation and immune phenotype alteration promote clonal evolution, dysregulate cancer signaling pathways activation and proteins expression, finally lead to venetoclax resistance

The regulation of apoptosis by BCL2 family proteins is achieved by the balance between pro-apoptotic proteins and anti-apoptotic proteins [4, 20]. The anti-apoptotic proteins include BCL2, BCL-XL, MCL1, BCL-w, and so forth. The pro-apoptotic proteins can be further divided into two subtypes according to their structure: the multidomain proteins, such as BAX and BAK; and the BH3-only proteins, such as BID, BIK, NOXA, PUMA, BAD, BIM (also known as BCL-XL1), and so forth [7]. The BH3-only protein is an apoptosis activator that can initiate cell apoptosis by inhibiting the anti-apoptotic proteins or directly activating the pro-apoptotic proteins [7]. BCL2 and its anti-apoptotic family members can bind to and inactivate BAX or BAK, or directly bind to BH3-only proteins to block apoptosis, so as to maintain cell survival and proliferation [21]. Under the influence of the death signal, BAX or BAK dissociates from BCL2, and the activated free BAX/BAK undergoes oligomerization and forms holes in the outer mitochondrial membrane, leading to the entry of cytochrome c and the other pro-apoptotic molecules in the mitochondria into the cytoplasm, and then activating the caspases, leading to the occurrence of apoptosis [20–23]. On the contrary, the overexpression of anti-apoptotic proteins MCL1 and BCL-XL blocks the activation of BAX/BAK by binding to BH3-only proteins, thereby blocking cell apoptosis. Several previous studies showed that the ratio of the expression levels of anti-apoptotic proteins and pro-apoptotic proteins often indicated sensitivity to venetoclax [5, 24, 25]. Therefore, the main molecular mechanism of venetoclax resistance is dysregulating of BCL2 family proteins and other non-BCL2 family proteins [20, 26, 27] caused by different reasons. This review aimed to summarize the mechanisms according to the biological processes and the combination therapy strategies to improve the efficacy of venetoclax. Figure 1 depicts potential mechanisms of venetoclax resistance.

## Mutation of target proteins

Mutation of the target proteins is pivotal in the failed treatment of cancers, which may change the binding sites of protein inhibitors and impeding functions of the affected proteins [28]. The BH3-binding groove of BCL2 protein is a venetoclax-binding site, whose mutations lead to the change in protein conformation, impeding venetoclax binding to BCL2 or downregulating the binding affinity, thereby inducing venetoclax resistance [26, 29–31]. After long-term in vitro induction, lymphoma cell lines LyBCL2-6, LyBCL2-9, and SC-1 were resistant to venetoclax. Sequencing of the BCL2 family protein gene showed that some missense mutations (F101C, F101L, or F104L) in the same codon within the BH3 domain of BCL2 were detected in the resistant cells, which were different from their parents’ sensitive cell lines [29, 30]. In patients with venetoclax-resistant CLL, whole-exome sequencing revealed acquiring variants of *BCL2*-G101V and *BCL2*-D103Y at different time points [26, 32]. Another advanced study showed *BCL2*-G101 mutation in venetoclax-resistant patients and confirmed it as a de novo acquisition mutation compared with pre-treatment samples [31, 33]. However, the frequency of mutation was relatively as low as 1%, implying other mechanisms of clonal shifts and resistance to venetoclax [33].

## Clonal evolution

Numerous cytogenetic and molecular genetic abnormalities in tumor cells often lead to resistance to venetoclax, although part of the pathological mechanism is still unclear. Previous studies on patients with AML treated with venetoclax combined with HMAs or LDAC showed that patients with nucleophosmin1 (*NPM1*) or isocitrate dehydrogenase 2 (*IDH2*) mutations had better responsiveness to venetoclax. Especially, patients with *NPM1* mutations often have longer molecular remission time. In addition, in patients with fms-like tyrosine kinase 3 (*FLT3*) internal tandem duplication gain, the clonal activation of the RAS pathway or tumor protein 53 (*TP53*) biallelically perturbation suggests resistance to venetoclax combined with HMAs or LDAC [34]. The upregulation of BCL2 anti-apoptotic gene expression caused by the activation of signaling pathways [mitogen-activated protein kinase (MAPK), extracellular-signal-regulated kinase (ERK), or phosphatidylinositol 3-kinase (PI3K)/AKT] in cells may be one of the mechanisms of drug resistance [35, 36]. Among patients with MCL treated with venetoclax combined with ibrutinib, patients with SWI-SNF chromatin-remodeling complex often have primary drug resistance or early recurrence. SWI-SNF complex-induced BCL-XL expression may be a mechanism of drug resistance [37]. A consensus has been formed regarding the existence of many subclones of tumor cells, and clonal evolution inevitably occurs during the development of the disease. Genetic abnormalities can appear in the early stages of the disease or during relapse and drug resistance.

Clonal evolutions are the final results of genome alterations. Ongoing clonal evolution of CLL cells under venetoclax treatment pressure is a crucial step in drug resistance. Hence, deciphering the detailed mechanisms can help gain an insight into the failure of venetoclax therapy and disease progression [38, 39]. Whole-exome sequencing and methylation profiles were used with the primary CLL samples collected from patients before venetoclax therapy to reveal the genome alterations and clonal shifts. The baseline of genome alteration was about 25.5 mutations before venetoclax treatment [38]. After exposure to venetoclax, nearly 12.5% of the genome changed when drug resistance occurred, indicating the CLL clonal evolution.

The patterns of clonal evolution are heterogeneous, including linear, convergent, and divergent. Somatic genome alterations showed abundant mutations and recurrent alterations. Genome focal amplification related to programmed death ligand 1 (PD-L1) expression was observed when patients developed venetoclax resistance, the site of amplification also including CD274-encoding region, which, after translation, induced a prominent infiltration of CD3-positive lymphocytes, indicating that the new subclone might be susceptible to immune therapy [38, 40]. Further, 3.8% of patients with venetoclax resistance acquired v-raf murine sarcoma viral oncogene homolog B1 (BRAF) mutation. BRAF mutation has been confirmed as oncogenic. Inducing mutant BRAF expression in CLL cell line OCY-LY19 upregulated MCL1 protein synthesis and increased the IC50 of venetoclax, indicating that it might be a driver mutation of CLL clonal evolution [38, 41].

A recent study reported that CLL clonal shifts drove the drug resistance, and the BCL2 family took part in the pathogenesis. Amplification of BCL2, MCL1, and BCL-XL in CLL when venetoclax therapy failed, and the overexpression of these genes in CLL cell line increased the IC50 of venetoclax [39]. In MCL, clonal evolution also drove drug resistance [42]. In patients resistant to ibrutinib, a combination of ibrutinib with venetoclax showed a synergistic effect; however, drug resistance was inevitable. Whole-exome sequencing showed SMARCA4 and KMT2C/D mutations as new genome alterations at progression. Harboring these two gene mutations might initiate de novo clone formation and venetoclax resistance [42].

## Tumor microenvironment

Chemo-resistance mediated by the tumor microenvironment is another major problem in cancer treatment [25, 43–45]. The lymph nodes and bone marrow are the main shelters of hematologic malignant cells, where a variety of cell components activate the signaling pathways on tumor cells and promote cancer progression [25, 43]. The activation of multiple signaling pathways in tumor cells that promote cell proliferation and survival, such as the activation of B-cell receptor (BCR) and its downstream pathway signaling molecules [45, 46] and the activation of the ERK pathway caused by the activation of extracellular receptors [36], can induce the upregulation of BCL2 family anti-apoptotic proteins, which is one of the mechanisms of venetoclax resistance caused by the microenvironment. Therefore, this may be the intracellular molecular mechanism of small-molecule inhibitors of various kinases combined with venetoclax in the treatment of hematologic tumors.

Activated T cells in the microenvironment can produce cytokines such as IL-4 and IL-21, which can stimulate CD40 on lymphoma cells and increase the expression of MCL1, BCL-XL, and BFL1. On co-culturing CLL with control 3T3 cells (transfected with empty vector) or 3T40L cells (CD-40 ligand-transfected NIH3T3 cells) in the presence of IL-4 or IL-21, CLL showed resistance to venetoclax compared with the control 3T3. In addition, knocking down BCL-XL in 3T40L cells could reverse the resistance phenomenon of venetoclax, thus confirming the role of BCL-XL in venetoclax treatment failure. Furthermore, on combining venetoclax with dasatinib, CD40-mediated venetoclax resistance was perturbed, indicating the synergistic effect of venetoclax and dasatinib [44].

In CLL, human bone marrow mesenchymal stem cells (HBMSCs) released extracellular vesicles to induce venetoclax by regulating gene expression profiles [47]. Co-culturing CLL cells with the supernatant of BMSCs could protect tumor cells from venetoclax-induced apoptosis [47]. A previous study reported that venetoclax and ibrutinib increased the expression of cleaved Poly (ADP-Ribose) Polymerase 1 (PARP1) and induced cell apoptosis synergistically [43]. However, in the presence of HBMSCs or microenvironment cytokines such as IL-10, CD40L, and CPG-ODN, the synergistic effect was reversed and drug resistance developed. Further molecular mechanisms were revealed; the activation of the NF-κB signaling pathway upregulated the expression of the BCL2 family members, which was proved to be the cornerstone of venetoclax resistance [48].

## Dysregulation of mitochondrial energy metabolism

Dysregulation of mitochondrial energy metabolism also takes part in venetoclax resistance. In CLL cell line OCI-Ly1 with amplification of 1q23, AMPK/PKA pathway was aberrantly activated, which perturbed cytochrome c release and finally led to venetoclax resistance [39]. Also, running out of ATP in the inner layer of mitochondrion could stimulate the AMPK/PKA pathway, showing a synergistic effect with the amplification of 1q23 [39]. Using a genome-wide CRISPR knockout screen, Sharon et al. [49] found that gene inactivation involving mitochondrial translation sensitized AML cells with drug resistance to venetoclax. The inhibition of mitochondrial respiration by inhibiting the electron transport chain (ETC) complex 1 is one of the mechanisms by which venetoclax kills AML cells, suggesting that mitochondrial energy metabolism disorders are involved in the resistance of venetoclax. The combined use of drugs, such as tedizolid, which target the mitochondrial respiratory chain through different mechanisms, can further enhance the anti-AML effect of venetoclax combined with azacitidine in vivo and in vitro [49, 50]. In leukemic stem cells, mutant *TP53* perturbed mitochondrial homeostasis by dysregulating transcription factor DP-1 (TFDP1) activation and translocation of phorbol-12-myristate-13-acetate-induced protein 1 (PMAIP1) into the mitochondria, impairing the effector function of BAX/BAK [51]. Moreover, *TP53* mutation also impedes BCL2 expression, decreasing the target of venetoclax directly and leading to drug resistance [51].

## Combination strategies

In view of the drug resistance mechanisms of venetoclax, researchers have designed a variety of combination treatment strategies to improve the clinical efficacy of venetoclax. In this section, the clinical research results of combined treatment in a variety of hematologic malignancies have been summarized and are also listed in Table 1. The ongoing clinical trials are listed in Table 2.

**Table 1:** Result of combination therapy strategy in hematologic malignancies

Combination therapy strategy	Disease state(s)	Efficacy	Reference or Clinicaltrials.gov identifier	Phase
Venetoclax + low-dose cytarabine	Previously untreated AML	ORR: 54% Median OS: 10.1 months Median DOR: 8.1 months	Wei et al. NCT02287233 [17]	Ib/2
Venetoclax + HAMs (decitabine or azacitidine)	Treatment-naïve AML	ORR: 68% (99/145) Median OS: 17.5 months Median DOR: 11.3 months MRD negative: 29% (28/97)	DiNardo et al. NCT02203773 [16]	Ib
Venetoclax + ibrutinib	Previously untreated high-risk and elderly CLL	ORR: 88% (29/33) Estimated 1-year PFS: 98% Estimated 1-year OS: 99% BM MRD negative: 61% (20/33)	Jain et al. NCT02756897 [72]	II
Venetoclax + rituximab	RR CLL	ORR: 86% (42/49) Estimated 2-year PFS: 82% Estimated 2-year OS: 89% BM MRD negative: 80% (20/25)	Seymour et al. NCT01682616 [74]	I
Venetoclax + rituximab vs bendamustine + rituximab	RR CLL	ORR: 93.3% vs 67.7% CR/CRi: 26.8% vs 8.2% 2-year PFS: 84.9% vs 36.3% 2-year OS: 91.9% vs 86.8% BM MRD negative: 27.3% vs 1.5%	Seymour et al. NCT02005471 [75]	III
Venetoclax + obinutuzumab	Previously untreated and RR CLL	RR and 1L patients ORR: 95% (41/43) and 100% (32/32) Estimated 24-month PFS: 85.4% and 90.6% Median DOR: 40.9 months and NR (not reached) BM MRD negative: 64% (26/42) and 78% (25/32)	Flinn et al. NCT01685892 [79]	Ib
Venetoclax + ibrutinib + obinutuzumab	RR CLL	ORR: 92% (11/12) Estimated 24-month PFS: 92% BM and PB MRD negative: 50% (6/12)	Rogers et al. NCT02427451 [76]	Ib
Venetoclax + R-CHOP/G-CHOP	RR NHL (DLBCL, FL)	ORR of all patients: 87.5% CR/CRi of R-CHOP and G-CHOP: 79.2% and 78.1% 1-year PFS of R-CHOP and G-CHOP: 70% and 100%	Zelenetz et al. NCT02055820 [93]	Ib
Venetoclax + bendamustine + rituximab	RR NHL (DLBCL, FL, and MZL)	ORR of all patients: 65% Median DOR of all patients: 38.3 months Median PFS of all patients: 10.7 months Median OS of all patients: not reached	De Vos et al. NCT01594229 [91]	Ib
Venetoclax + ibrutinib	RR or previously untreated MCL	ORR: 71% (17/24) 12-month estimated PFS: 75% 12-month estimated OS: 79% BM MRD negative: 67% (16/24)	Tam et al. NCT02471391 [102]	II
Venetoclax + bortezomib + dexamethasone	RR MM	ORR: 67% (44/66) Median DOR: 9.7 months Median TTP: 9.5 months	Moreau et al. NCT01794507 [110]	Ib

*AML* acute myeloid leukemia, *BM* bone marrow, *CLL* chronic lymphocytic leukemia, *CR* complete remission, *CRi* complete remission with incomplete count recovery, *DLBCL* diffuse large B-cell lymphoma, *DOR* duration of remission, *FL* follicular lymphoma, *HMA* hypomethylating agent, *NHL* non-Hodgkin's lymphoma, *MCL* mantle cell lymphoma, *MM* multiple myeloma, *MRD* minimal residual lesion, *ORR* objective remission rate, *OS* overall survival, *PFS* progression-free survival, *RR* relapsed/refractory, *TTP* time to progression

**Table 2:** Ongoing and planned combination clinical trials involving venetoclax

Combined drugs	Disease state(s)	Clinicaltrials.gov identifier	Phase
Azacitidine + venetoclax	Elderly, previously untreated AML	NCT03466294	II
LDAC + venetoclax	Treatment-naïve AML	NCT02287233	I/II
LDAC + venetoclax vs LDAC + placebo	Elderly; treatment-naïve AML	NCT03069352	III
Cobimetinib (MEK inhibitor) + venetoclax Idasanutlin (HDM inhibitor) + venetoclax	Elderly RR AML	NCT02670044	I
Cobimetinib (MEK inhibitor) + venetoclax Idasanutlin (MDM inhibitor) + venetoclax	RR AML	NCT02670044	I
Dinaciclib + venetoclax	RR AML	NCT03484520	I
Alvocidib + venetoclax	RR AML	NCT03441555	I
Gilteritinib (FIT3 inhibitor) + venetoclax	RR AML	(NCT03625505)	I
Quizartinib (FIT3 inhibitor) + venetoclax	*FLT3* mutated RR AML	(NCT03735875)	Ib/II
S64315 + venetoclax	AML	NCT03672695	I
AZD5991 + venetoclax	RR AML or MDS	NCT03218683	I/II
CYC065 (CDK inhibitor) + venetoclax	RR AML or MDS	NCT04017546	I
Alvocidib (flavopiridol) + venetoclax	RR AML	NCT03441555	I
Obinutuzumab + venetoclax	RR or previously untreated CLL	NCT01685892	I
Ibrutinib + obinutuzumab + venetoclax	CLL	NCT03755947	II
Acalabrutinib (BTK inhibitor) + venetoclax	Newly diagnosed CLL	NCT03868722	II/III
Acalabrutinib + obinutuzumab + venetoclax	CLL	NCT03580928	II
Zanubrutinib (BTK inhibitor) + obinutuzumab + venetoclax	Previously untreated CLL or SLL	NCT03824483	II
Ublituximab + umbralisib + venetoclax	CLL	NCT03801525	II
Duvelisib (PI3K inhibitor) + venetoclax	RR CLL or SLL or RS	NCT03534323	I/II
Obinutuzumab + venetoclax	RR DLBCL	NCT02987400	II
Ibrutinib + rituximab + venetoclax	RR DLBCL	NCT03136497	I
Obinutuzumab + polatuzumab vedotin (ADC against CD79b) + venetoclax	RR DLBCL or FL	NCT02611323	I
Rituximab + venetoclax	RR FL	NCT02187861	II
Obinutuzumab + ibrutinib + venetoclax	RR MCL	NCT02558816	I/II
Ibrutinib + venetoclax	RR MCL	NCT02471391	II
Ibrutinib + venetoclax vs ibrutinib + placebo	MCL	NCT03112174	III
Carfilzomib + dexamethasone + venetoclax	RR MM	NCT02899052	II
Bortezomib + dexamethasone + venetoclax vs bortezomib + dexamethasone + placebo	RR MM	NCT02755597	III
Pomalidomide + dexamethasone + venetoclax	RR MM	NCT03567616	II

*ADC* antibody–drug conjugate, *AML* acute myeloid leukemia, *BTK* Bruton’s tyrosine kinase, *CDK* cyclin-dependent kinases, *CLL* chronic lymphocytic leukemia, *DLBCL* diffuse large B-cell lymphoma, *FL* follicular lymphoma, *HDM2* human double minute 2, *LDAC* low-dose cytarabine, *MDS* myelodysplastic syndrome, *MEK* mitogen-activated protein kinase kinase, *MCL* mantle cell lymphoma, *MM* multiple myeloma, *PI3K* phosphatidylinositide 3-kinase, *RR* relapsed/refractory, *RS* Richter's syndrome, *SLL* small lymphocytic lymphoma

## Acute myeloid leukemia

AML is a disease caused by the blocked differentiation of myeloid hematopoietic stem cells and the clonal proliferation of primitive or immature myeloid cells in the bone marrow. A phase II trial confirmed that the ORR of venetoclax monotherapy was only 19% (6/32) in patients with RR AML or previously untreated AML who were not suitable for intensive chemotherapy [15]. However, patients with AML having *IDH1/2* mutations may have better efficacy, with complete remission or complete remission with incomplete count recovery (CR/CRi) of 33% [52].

Previous studies showed that the efficacy of venetoclax clearly improved when combined with drugs that downregulated MCL1 and/or BCL-XL. HMAs and cytarabine can downregulate the expression of MCL1, and may exert a synergistic effect with venetoclax to interfere with the energy metabolism of AML stem cells and kill tumor cell [50, 53, 54]. A phase Ib/II trial investigated the efficacy of venetoclax in combination with LDAC in 82 elderly patients with AML who were not eligible for intensive chemotherapy. The CR/CRi was 54%, and the patients with de novo AML, intermediate-risk cytogenetic features, and without prior HMA exposure had the highest rates of CR/CRi (71%, 63%, and 62%, respectively). The median overall survival (OS) for all patients was 10.1 months, and the median duration of remission (DOR) was 8.1 months. Bone marrow suppression was the most common adverse event (AE) [17] (Table 1). In a phase Ib study, 57 patients with newly diagnosed AML who were not suitable for intensive chemotherapy were randomly divided into three groups. Group A received combined treatment with venetoclax and decitabine, and group B received combined treatment with venetoclax and azacitidine; the total ORR of the two groups was 62%, and the CR/CRi was 60%. The ORR of groups A and B was 65% and 59%, respectively. The median DOR of the two groups was 11.0 months, and the median OS was 15.2 months. Hematologic toxicity was the most common adverse reaction [55] (Table 1). In a phase Ib trial, the efficacy of venetoclax in combination with decitabine or azacitidine was evaluated in 145 treatment-naïve AML ineligible for intensive chemotherapy; 49% of the patients had cytogenetic abnormalities with poor prognosis. After a median of 8.9 months of treatment, 67% of the patients achieved CR/CRi; among the patients with CR/CRi, 29% of patients were minimal residual disease (MRD) negative. The median OS was 17.5 months, and the estimated 2-year OS was 46%. Hematological and gastrointestinal AEs were the most common toxicities observed [16] (Table 1).

Small-molecule kinase inhibitors can downregulate the expression of BCL2 family apoptosis inhibitors through the regulation of cell signaling pathways [35, 36]. Cobimetinib (GDC-0973), an allosteric MEK inhibitor, was demonstrated to have synergistic anti-leukemic effect with venetoclax in vitro by inhibiting the proliferation of AML cell lines and primary AML cells and reducing the leukemic burden in xenograft mouse models [35]. A phase I clinical trial of the combination of venetoclax and cobimetinib is currently being conducted on patients with RR AML (NCT02670044) (Table 2). In patients with AML having *FLT3* mutations, the corresponding kinase inhibitors can also be used in combination [20]. For example, sorafenib inhibits *FLT3* and downregulates MCL1 expression. It can synergize anti-AML with venetoclax. However, clinical trials are still needed to confirm its efficacy [56, 57]. The safety and scientific validity of venetoclax in combination with *FLT3* inhibitors gilteritinib (NCT03625505) and quizartinib (NCT03735875) are currently being tested in RR AML with *FLT3* mutation (Table 2).

The *TP53* gene is an important tumor suppressor gene that regulates apoptosis and cyclin expression. The mutations or deletions of the *TP53* gene are associated with the occurrence and development of a variety of tumors, which can downregulate the expression of BCL2 family pro-apoptotic proteins in AML cells, leading to the inactivation of the mitochondrial apoptotic pathway [8, 58]. Murine double minute homolog 2 (MDM2) is the most important negative regulator of *TP53*. The combination of MDM2 inhibitor idasanutlin and venetoclax may significantly inhibit the proliferation and induce apoptosis of wild-type AML cells (OCI-AML3) in vitro and in vivo. The cell line has high expression of MCL1 and is resistant to both idasanutlin and venetoclax [59]. A phase I clinical trial is currently testing the tolerability and safety of venetoclax in combination with cobimetinib or idasanutlin in patients with RR AML who are not suitable for cytotoxic therapy (NCT02670044) (Table 2).

A direct and effective treatment strategy to overcome venetoclax resistance is to combine it with specific MCL1 inhibitors [23, 60, 61]. A-1210477 is the first high-affinity, selective MCL1 inhibitor. It can synergistically inhibit the proliferation of BCL2/MCL1-dependent AML cell lines and induce apoptosis when combined with venetoclax [61]. VU661013, a novel, potent, selective MCL1 inhibitor, which destabilizes the BIM/MCL1 association, leads to apoptosis in AML and is active in venetoclax-resistant cells and patient-derived xenografts. It can synergize with venetoclax to kill AML cells [23]. S63845 is a selective MCL1 inhibitor that can selectively bind to the BH3-binding groove of MCL1, thereby effectively killing MCL1-dependent tumor cells, including MM, leukemia, and lymphoma cells [60]. A phase Ib clinical trial (NCT03672695) combining venetoclax with the selective MCL1 inhibitor S64315 is currently underway on patients with AML (Table 2).

## Acute lymphoblastic leukemia

Acute lymphoblastic leukemia (ALL) is a malignant neoplastic disease in which lymphocytic B or T cells proliferate abnormally in the bone marrow, and can also invade extramedullary tissues. In vitro studies indicated that MCL1-specific inhibitor S63845 and venetoclax could synergistically suppress T-ALL cells, but the two drugs had no independent killing effect on T-ALL cells, which might be due to the high expression of MCL1 and BCL2 in T-ALL cells [62]. Animal experiments on ALL cell xenotransplantation confirmed the killing effect of venetoclax on ALL cells, especially in patients with mixed-lineage leukemia (MLL)-rearranged leukemia. However, in ALL cells, the expression level of BCL-XL largely determines the sensitivity of ALL cells to venetoclax [63]. Animal experiments on ALL cell xenotransplantation confirmed the killing effect of venetoclax on ALL cells, especially in patients with mixed-lineage leukemia (MLL)-rearranged leukemia. However, in ALL cells, the expression level of BCL-XL largely determines the sensitivity of ALL cells to venetoclax [64]. In Philadelphia chromosome-positive ALL, tyrosine kinase inhibitor (dasatinib and ponatinib) can upregulate BIM and inhibit the expression of MCL1, thereby cooperating with venetoclax to inhibit ALL cells [65]. However, the aforementioned conclusions still need to be carefully verified in patients.

## Chronic lymphocytic leukemia

CLL is a disease involving the clonal proliferation of mature B lymphocytes. Previous studies showed that almost all CLL cells overexpressed BCL2 [66]. An exploratory clinical study showed that venetoclax monotherapy had a certain therapeutic effect in patients with CLL, with an ORR of 80% and a CR of 40% in patients with newly diagnosed CLL [67]. Among patients with high-risk RR CLL, the CR/CRi was only 8%, although an ORR of 79% was achieved [11], suggesting that some CLL cells were resistant to venetoclax. Previous studies showed that the combination of anti-CD20 monoclonal antibodies (rituximab and obinutuzumab) and some kinase inhibitors, such as the combined use of TKIs, spleen tyrosine kinase inhibitors, BTK inhibitors, and phosphatidylinositol 3-kinase (PI3K) inhibitors, can overcome resistance to venetoclax, which can effectively eliminate the upregulation of anti-apoptotic genes in the BCL2 family, such as MCL1, BCL-XL, and BFL-1/A1, caused by the activation of microenvironment-mediated and intracellular signaling pathways, thereby overcoming venetoclax resistance [44–46, 68–71].

Venetoclax and the BTK inhibitor ibrutinib have a synergistic effect [69, 70]. Ibrutinib-mediated BTK inhibition can lead to a decrease in MCL1 protein expression, while it increases or has no effect on BCL2 levels. In addition, ibrutinib can effectively mobilize CLL cells from the microenvironment that provides growth signals, avoiding venetoclax resistance mediated by the tumor microenvironment [44, 71]. A phase II study explored the effect of ibrutinib combined with venetoclax, involving previously untreated high-risk and older patients with CLL. After 18 cycles, CR/CRi was as high as 96%, and 69% of patients had remission with undetectable MRD in the bone marrow. The estimated 1-year PFS and OS were 98% and 99%, respectively [72] (Table 1), suggesting that venetoclax combined with ibrutinib had a better effect in patients with high-risk and early-stage CLL.

Venetoclax in combination with an anti-CD20 monoclonal antibody has been shown to overcome microenvironment-mediated resistance to venetoclax [44]. Rituximab can reduce the expression of MCL1 protein and increase the sensitivity of CLL cells to venetoclax-induced apoptosis [73]. In a phase I study, venetoclax in combination with rituximab was administered to patients with RR CLL. Overall, 86% of the patients achieved ORR, including 51% with CR/CRi. Negative MRD in the bone marrow was achieved in 80% of CR/CRi responders and 57% of all patients. The estimated 2-year PFS and OS were 82% and 89%, respectively. AEs appeared to be similar to venetoclax in monotherapy [74] (Table 1). Another phase III MURANO trial compared the efficacy of venetoclax combined with rituximab (194) and bendamustine combined with rituximab (195) in patients with RR CLL. The results suggested that the ORR was 92.3% and CR/CRi was 26.8% in the venetoclax group, which were significantly higher than those (72.3% and 8.2%, respectively) in the bendamustine group. The evaluable peripheral blood MRD-negative rate in the venetoclax and bendamustine groups was 62.4% and 13.3%, respectively. In addition, the 2-year PFS rate (84.9%) and the 2-year OS rate (97.9%) were also significantly higher in the venetoclax group than in the bendamustine group. Even in the high-risk group of del17p patients, venetoclax had obvious advantages, and the 2-year PFS was 81.5% and 27.8%, respectively [75] (Table 1), suggesting that the combination of venetoclax and rituximab was effective in patients with RR CLL.

Obinutuzumab is an artificial, glycosylated type II anti-CD20 monoclonal antibody with excellent single-agent activity in CLL [76, 77],, and its efficacy is superior to that of rituximab [78]. In a phase Ib trial, venetoclax combined with obinutuzumab was administered for six cycles, followed by venetoclax monotherapy until disease progression (R/R) or two-drug combination treatment was administered for a fixed duration of 1 year (1L). Overall, ORR was 95% and 100% in R/R and 1L patients, respectively, and 37% of the R/R patients and 78% of the 1L patients achieved CR/CRi. The rate of undetectable MRD in the bone marrow for R/R and 1L patients was 64% and 78%, respectively. The estimated 24-month PFS was 85.4% and 90.6% in R/R and 1L patients, and the median DOR was 40.9 months and not reached, respectively. The most common grade 3–4 AE was neutropenia [79]. This experiment confirmed that the combination of venetoclax and obinutuzumab was effective and had acceptable therapeutic toxicity.

The combination of venetoclax with kinase inhibitors and anti-CD20 monoclonal antibodies may lead to better outcomes. A phase Ib trial investigated the efficacy and safety of the combined use of venetoclax, ibrutinib, and obinutuzumab in patients with RR CLL. Despite only 12 patients with RR CLL, all patients completed 14 cycles of treatment. ORR was up to 92% in the early evaluation, with 42% of the patients achieving CR/CRi. All patients achieved MRD undetectable in either the blood or the bone marrow, with 50% of the patients achieving MRD undetectable in both the blood and the bone marrow. The estimated 24-month PFS was 92%. Hematologic toxicity was the most frequent AE [76] (Table 1).

## Diffuse large B-cell lymphoma

Diffuse large B-cell lymphoma (DLBCL) is the most common aggressive NHL [80]. Rituximab, cyclophosphamide, doxorubicin, vincristine, prednisone (R-CHOP) is the first-line immunochemotherapeutic regimen for patients with newly diagnosed DLBCL. Although its CR could reach 76%, still 20–40% of patients failed to treatment or relapsed [81, 82] no effective regimen was recommended for these patients. In phase I clinical trial, venetoclax monotherapy was administered to patients with RR DLBCL. The ORR was only 18%, the CR was only 12%, and the estimated median PFS was only 1 month [13], suggesting that venetoclax monotherapy had limited activity and short-term effects in DLBCL. This might be related to the activation of multiple signaling pathways, such as Akt, in DLBCL tumor cells, leading to the overexpression of BCL2 family anti-apoptotic proteins [83–85]. Previous studies showed that a variety of kinase inhibitors, such as BTK inhibitors (ibrutinib), SYK inhibitors (R406), PI3K/mTOR dual inhibitors (NVP-BEZ235), PI3K inhibitors (idelalisib, copanlisib, ACP-319, and KA2237) or ATK inhibitors (MK-2206), and CDK9 inhibitor (dinaciclib) [86, 87], alone or in combination with venetoclax, significantly reduced the expression of anti-apoptotic proteins BCL2 tumor cells in vitro and then exerted synergistic killing effects on lymphoma cells. This conclusion was further confirmed in patient-derived xenograft (PDX) animal models [83, 86, 88]. Some of them are undergoing clinical trials to evaluate the effect of combined application with venetoclax.

Homoharringtonine (HHT) is a commonly used anti-leukemia drug with multiple mechanisms of action, including the downregulation of MCL1 expression [89]. Previous studies showed that HHT combined with venetoclax in DLBCL cell lines and mouse models of xenografts produced synergistic effects, suggesting that this combination might be a promising combination therapy [90]. However, corresponding clinical application data are still lacking.

Preclinical studies confirmed that venetoclax in combination with bendamustine and rituximab (BR) could exhibit synergistic effects. A phase Ib clinical trial enrolled 60 patients with RR NHL [32 with follicular lymphoma (FL), 22 with DLBCL, 6 with MZL] receiving venetoclax and BR combination therapy. After the median follow-up of 7.5 months, the ORR of all patients was 65% and the CR was 30%. Among them, the ORR of patients with DLBCL was 41% and the CR was 14%. Despite having a slightly lower effect in patients with FL and MZL, the combination was better than venetoclax as a single agent [91] (Table 1). Previous studies confirmed that the R-CHOP treatment regimen was less beneficial against BCL2-positive DLBCL [92]. Therefore, the use of BCL2 inhibitors in combination with R-CHOP in such patients may have beneficial effects. A phase Ib/II CAVALLI rial enrolled 56 patients with newly diagnosed DLBCL (including 43% FL and 32% DLBCL) receiving venetoclax in combination with R/G-CHOP therapy. The total ORR was 87.5%, of which the ORR of patients with DLBCL was 88.9%. These patients achieved CR, and the CR of patients with DLBCL having a dual expression of BCL2 and MYC was 87.5% [93] (Table 1).

## Follicular lymphoma

FL is one of the most common indolent lymphomas. Studies showed that 85–90% of patients with FL had a t(14;18) translocation, resulting in the overexpression of BCL2 [94, 95]. However, in a phase I clinical trial, the ORR of venetoclax monotherapy in patients with RR FL was 38%, and the CR was only 14%. The estimated median PFS was 11 months [13]. Therefore, even if FL is a disease characterized by high expression of BCL2, the effect of venetoclax monotherapy is still limited. The overexpression of BCL2 family anti-apoptotic proteins caused by the activation of multiple signaling pathways, such as JNK, AKT and ERK1/2 pathways in tumor cells, is the main mechanism of drug resistance [24]. Therefore, a combination with specific ERK inhibitors, pan-PI3K inhibitors, rituximab, and so forth, could significantly enhance venetoclax-induced FL cell apoptosis and overcome venetoclax resistance [24].

Preclinical studies confirmed that venetoclax combined with BR could lead to 100% complete tumor regression in NHL xenograft models with t(14;18) translocation [91]. In a phase Ib clinical trial, the use of venetoclax in combination with BR was investigated in patients with RR FL. After a 7.5-month follow-up period, the ORR was 75%, the CR was 38%, and the PR was 38%, which was significantly higher than that in DLBCL [91] (Table 1). In the aforementioned CAVALLI study, 24 patients with FL received venetoclax combined with R/G-CHOP and achieved an ORR of 83.3%; CR was 75%, PR was 8.3%, and the 1-year PFS was more than 90% [93] (Table 1).

## Mantle cell lymphoma

MCL is an aggressive small B-cell lymphoma, usually overexpressing BCL-2 [67]. In a phase I clinical trial, patients with RR MCL were treated with venetoclax monotherapy. The ORR was 75% and CR was only 14%; it was one of the best responding groups among patients with NHL [13]. Although venetoclax monotherapy is effective in patients with MCL, resistance to venetoclax is inevitable. Previous studies confirmed that the expression of MCL1 and BCL-XL could determine the sensitivity of MCL cells to venetoclax resistance [25, 96, 97]. Obinutuzumab has been proved to block the expression of BCL-XL by inhibiting NF-κB signaling in vitro, thereby counteracting the protective effect of the microenvironment and overcoming resistance to venetoclax in MCL cell lines [98].

Ibrutinib has been approved by the FDA for treating MCL [99]. It promotes MCL cells to enter the peripheral circulation by inhibiting chemokine and BCR signals and downregulating the expression of MCL1 in MCL cells, thereby overcoming tumor microenvironment-related drug resistance [100, 101]. A phase II clinical study evaluated the efficacy of venetoclax combined with ibrutinib in patients with RR or previously untreated MCL. Half of the patients had abnormal *TP53*, and 75% of the patients were high-risk patients. After 16 weeks of treatment, PET/CT assessment showed a CR of 67%, and 67% of the patients tested negative for MRD using flow cytometry. The median PFS was not reached, and the 12-month estimated PFS and OS rates were 75% and 79%, respectively. The most common AE was gastrointestinal toxicity [102] The combination of venetoclax with obinutuzumab and ibrutinib might be more effective than other combinations. Phase I/II trials are underway to assess the efficacy and safety of this combination in patients with RR MCL (NCT02558816) (Table 2).

Previous studies showed that venetoclax combined with the selective MCL1 inhibitor S63845 had a significant synergistic killing effect on MCL cell lines in vitro and induced long-term lymphoma-free survival in MCL xenograft models [103]. Therefore, venetoclax in combination with highly selective MCL1 inhibitors might be a promising treatment option in the future.

## Multiple myeloma

MM is a malignant proliferative disease of plasma cells. Previous studies showed that MM cells depended on the anti-apoptotic proteins BCL2, MCL1, and BCL-XL to survive [104, 105]. Venetoclax therapy is considered to be limited to subtypes of t(11;14) [14]. In a phase I clinical trial, venetoclax monotherapy was administered to patients with RR MM; the ORR was 21%, with 15% reaching VGPR or better. In the subset of patients with t(11;14) translocation, the ORR was 40%, and 27% achieved VGPR or better [14, 106].

Venetoclax monotherapy has limited efficacy in patients with MM. The overexpression of MCL1 is one of the important reasons for the inherent resistance of MM cells to venetoclax [105]. Therefore, the inhibition of MCL1 could increase the sensitivity of MM cells to venetoclax [107]. The proteasome inhibitor bortezomib can inhibit MCL1 indirectly by stabilizing the MCL1-neutralizing protein NOXA and overcoming the resistance of MM cells to venetoclax in a xenograft model [108]. Dexamethasone can upregulate the expression of BIM to increase the dependence of MM cells on BCL2, thereby increasing the sensitivity to venetoclax [109]. phase I clinical trial showed that the combined treatment of venetoclax and dexamethasone for patients with RR MM resulted in a significantly higher ORR (65%) compared with venetoclax monotherapy (40%) [7]. A phase Ib trial studied the efficacy of venetoclax in combination with bortezomib and dexamethasone in patients with RR MM. The ORR was 67%, with 42% achieving VGPR or better. In patients with and without t(11;14) translocation, the ORR rate was 78% and 65%, respectively. The ORR in patients with high and low expression of BCL2 was 94% and 59%, respectively. The median time to progression and DOR were 9.5 months and 9.7 months, respectively. The common AEs included mild gastrointestinal toxicities and grade 3/4 cytopenias [110] (Table 1), indicating that this three-drug combination regime had a good effect and mild adverse reactions.

CDK9 inhibitors, such as flavopiridol [111], seliciclib [112], and so forth, can also downregulate the expression of MCL1. Hence, when combined with venetoclax, these inhibitors can also produce a synergistic effect to overcome the resistance of MM to venetoclax. The efficacy of venetoclax in combination with the CDK9 inhibitors dinaciclib (NCT03484520) and alvocidib (NCT03441555) is currently being evaluated in patients with RR AML (Table 2).

## Conclusion

Venetoclax showed significant activity in hematologic malignancies, such as CLL, AML, MM, DLBCL, MCL, FL, and so forth. The occurrence and development of malignant tumors often involved multiple signaling pathways. Hematologic malignancies often developed acquired or inherent resistance to venetoclax. The most common mechanism of venetoclax resistance was the overexpression of the BCL2 family anti-apoptotic proteins, such as MCL1 and BCL-XL, for a variety of reasons. Based on this resistance mechanism, various clinical trials have been conducted to overcome resistance to venetoclax in recent years. Venetoclax-based combination regimens are important treatment options for the treatment of hematologic malignancies.

## Data Availability

Not applicable.

## Abbreviations

AEs: Adverse events
ALL: Acute lymphocytic leukemia
AML: Acute myeloid leukemia
BCL2: B-cell lymphoma-2
BCR: B-cell receptor
BM: Bone marrow
BRAF: V-raf murine sarcoma viral oncogene homolog B1
BTK: Bruton’s tyrosine kinase
CDK: Cyclin-dependent kinase
CLL: Chronic lymphocytic leukemia
CR: Complete remission
CRi: Complete remission with incomplete count recovery
DLBCL: Diffuse large B-cell lymphoma
DOR: Duration of remission
ERK: Extracellular-signal-regulated kinase
ETC: Electron transport chain
FDA: Food and Drug Administration
FL: Follicular lymphoma
FLT3: FMS-like tyrosine kinase 3
HBMSCs: Human bone marrow mesenchymal stem cells
HMAs: Hypomethylating agents
IDH2: Isocitrate dehydrogenase 2
LDAC: Low-dose cytarabine
MAPK: Mitogen-activated protein kinase
MCL1: Myeloid cell leukemia-1
MDM2: Murine double minute homolog 2
MEK: Methyl ethyl ketone
MLL: Mixed-lineage leukemia
MM: Multiple myeloma
MRD: Minimal residual disease
NHL: Non-Hodgkin's lymphoma
NPM1: Nucleophosmin1
ORR: Objective remission rate
OS: Overall survival
PD-L1: Programmed death ligand 1
PDX: Patient-derived xenograft
PFS: Progression-free survival
PI3K: Phosphatidylinositide 3-kinase
PMAIP1: Phorbol-12-myristate-13-acetate-induced protein 1
RR: Relapsed/refractory
R-CHOP: Rituximab, cyclophosphamide, doxorubicin, vincristine, prednisone
SYK: Spleen tyrosine kinase
TFDP1: Transcription DP-1
TLS: Tumor lysis syndrome
TKI: Tyrosine kinase inhibitor
TP53: Tumor protein 53
VGPR: Very good partial remission
